# On Scurvy

**Published:** 1816-04

**Authors:** T. Trotter


THE LONDON

### Medical and Physical Journal.

4 OF VOL. XXXV.] APRIL, 1816. [NO. 206.

"For many fortunate discoveries in medicine, and for the detection of numerous errors, the world is indebted to the rapid circulation of Monthly "Journals; and there never existed any work to which the Faculty in "Europe and America were under deeper obligations than to the "Medical and Physical Journal of London, now forming a long, but an "invaluable, series."—Rush.

*For the London Medical and Physical Journal.*

### *On Scurvy;* by T. Trotter, M.D.

A third edition of my "Observations on Scurvy" has, for some time, been called for; but it was thought advisable to delay it till the conclusion of hostilities, when the whole experience obtained during the last twenty years of war might be brought into one view. It will, therefore, oblige me, by making your pages the medium of conveying my intention to such naval medical officers as may wish to favour me with their correspondence on the Prevention and Cure of Scurvy.

This may be the last opportunity which may ever be offered to me of engaging in a professional enquiry connected with public service, and I should like to do it all the justice in my power. I shall ever bless Divine Providence for having placed me in the station of Physician to the Fleet when the general scurvy appeared, in every ship on the home station, in the severe winter of 179$\frac{4}{5}$. It then fell to my lot to see my own precepts exemplified on a larger scale of practice than may again happen to any succeeding physician. From this period are we to date the *total extirpation* of scurvy from his Majesty's ships; and while I do justice to my own official duty, with much pleasure I shall record the faithful exertions of every naval officer, and I can never sufficiently praise the unwearied zeal and ability of the surgeons on this arduous occasion.

At this time, the scene at Spithead was highly interesting, as well from its effects as from its novelty. After the most disgusting opposition from particular departments of office, I at last obtained every article in full quantity which I demanded. Lemons and oranges were sent to us by *express* in in light waggons; and a supply of forty hundred-weight of *fresh salad* was obtained daily in the immediate neighbourhood of Portsmouth. Cruizing squadrons were supplied for their expected time at sea; and not a single ship was rendered inactive. From the nature of the service, in which a great part of the fleet was constantly detached upon, it was seven months before the *taint* of scurvy could be said to be fairly eradicated; during which time, upwards of 30,000 cases underwent the ordeal of cure, in their own ships, and without a single death! For the first time, a British fleet beheld the ceremony of an hospital ship firing a gun at noon, to dispense the stores of health to a scorbutic navy.

From this time large tracts of ground, near the naval ports, were converted into gardens; and I received the discretional power from the Admiralty to order any quantity of vegetables in season for ships returning from long cruizes. A few years after this occurrence, the elegant preparation of the lemon by Mr. Coxwell, of Temple Bar, was put to the test of experiment, and found to answer all the purposes of the fresh lemon in the cure of scurvy. It would have afforded me great pleasure to have contributed to Mr. Coxwell's appointment as a naval chemist, in order to have superseded by his Concrete Acid every other form, as being easily portable, and liable to no changes by keeping;* but Lord Howe was now dead, and with him seems to have set the sun of improvement in the medical department of the royal navy.

Without some arrangements of the kind mentioned above, it would have been utterly impracticable for our fleets to keep the sea for the length of time often done; or to have sustained the incessant and laborious duty of a general blockade. In the home seas, and in cold climates, the scurvy is chiefly prevalent. If we compare the extent of our naval armaments for the last twenty years, with what we have seen and heard of former wars, at a moderate calculation, not less than the lives of 80,000 seamen have been saved in scurvy only, by these changes. Besides, the strength of body and muscular power were preserved, for wielding with effect a heavy artillery in the day of battle; for it is observed in this disease, that a brave man becomes a physical coward. Thus were our ships kept full of men,

* Every ship and vessel going out on foreign voyages should carry with them this *Concrete Acid of Lemon.* I recommended it long ago to the India Directors; but I am much afraid their ships still continue to suffer from scurvy.

while while our merchant vessels were little molested by impressing, compared with former times. It is, therefore, of much importance that our successors should be informed of the horrors which the service has escaped from, that it may avoid such evils in the event of future wars.

The years 1794–5 form an epoch in the medical history of the British navy. A contagion, spread from the French prisoners taken on the 1st of June, the most extensive of the kind ever known in a fleet, was subdued without exciting the smallest alarm in the country; while the means of safety were rendered familiar to the officer, and interwoven with permanent discipline. The prevention and cure of scurvy were fixt on an establishment for the supply of vegetables and fruits in season, as permanent naval stores. The royal hospital were surveyed, and searched in every department; and a new system introduced. The improvement of medical pay was begun. Regular sick apartments, with bedding and utensils, &c. were first granted. Comforts for the use of the sick in their own ships were improved to the utmost. The hospital ship of the fleet was new modelled: it carried to sea, besides the ship's provisions, all the fruits and vegetables in season; live-stock; pickles; Seville oranges made into marmalade; Port and Lisbon wine; porter; cyder; tea, coffee, cocoa; sugar; eggs for puddings, &c.; and in 1797 a milch cow was embarked. To these may be added, the abolition of the disgraceful fine of 15s. levied on the seaman, and paid to the surgeon, for the cure of the venereal disease. On the whole, compared with former times, the sick-bed of the seaman was made a bed of comfort.

I must embrace the opportunity now afforded me in this address, to remind the profession, that, during the last twenty years of war, it has been the lot of our fleets and armies to be employed in seas and countries where a British force was never before known. Now the history of health in these situations must have exhibited many facts of importance, as well as novelty. Would it not, therefore, be worthy of the glory which our arms have acquired, to preserve the knowledge thus obtained from sinking to oblivion? Having thrown out this hint for others to act upon, with much confidence I look to Sir James M'Gregor, at the head of the army medical department, for its completion; and my friend Robert Jackson, who has just returned from another Anson-voyage, on the fevers of the West Indies.

The British navy has again to bewail the direction of its medical department passing to another of the inferior boards. If the Admiralty could not find medical officers in the navy qualified to form a board of science, to watch the duties of the sick bed; it might have spared the service the mortification of seeing its surgeons put under the command of a *few superannuated pursers,* by taking the appointments and patronage under its own wing. What member of the profession but must view these transfers with disgust! No wonder that an officer of my rank should have been followed to retirement by the *persecution of office*, while such a spirit continues to direct the navy.

I must beg my correspondents will be kind enough to *frank* their communications, for I have incurred an enormous expence from this cause since I retired.

*Newcastle; March,* 1816.

---

